# Visual impairment among eye health workers in a tertiary eye centre in Ghana

**DOI:** 10.4314/gmj.v55i4.8

**Published:** 2021-12

**Authors:** Naamuah N Tagoe, Benjamin Abaidoo, Gladys Fordjuor, Yakubu A Seidu, Serwaa A Acquah, Andrew E Akafo, Eileen Buxton, Dorothy Fiadoyor, George Afenyo, Samuel O Asiedu, Vera A Essuman

**Affiliations:** 1 Eye Centre, Korle Bu Teaching Hospital (KBTH), Accra; 2 Ophthalmology Unit, Department of Surgery, University of Ghana Medical School, College of Health Sciences, University of Ghana, Accra

**Keywords:** visual impairment, glaucoma, avoidable blindness, refractive errors

## Abstract

**Objective:**

To determine causes of visual impairment (VI) among staff of the Eye Centre at the Korle Bu Teaching Hospital.

**Design:**

This was a cross-sectional study.

**Setting:**

The Eye Centre, Korle Bu Teaching Hospital (KBTH), from October 2016 to March 2017 on all consenting members of staff.

**Participants:**

Eighty-four (79.3%) of 106 consenting staff members participated in this study.

**Data collection/Intervention:**

A detailed history (demographic, ocular, medical co-morbid conditions), ocular examination and relevant diagnostic investigations were conducted. Interventions initiated included treatment for glaucoma, dry eye and allergic conjunctivitis and spectacles prescription for refractive errors.

**Main outcomes:**

Prevalence of avoidable causes of VI (glaucoma, cataract, refractive errors). Secondary outcomes included prevalence of unavoidable causes of VI.

**Results:**

Eighty-four (79.3%) members of staff participated in this study. Most of the participants were females, 54(64.3 %). Age ranged from 23 to 60 years with an average of 35.8±9.9 years (mean ± SD).

Prevalence of VI was 9.5 % (8/84), all due to uncorrected refractive error. Other known causes of VI included open angle glaucoma in 12(14.3 %), macular scar of unknown cause, 1(1.2 %) and sutural cataract, 1(1.2 %) but were all visually insignificant.

**Conclusions:**

The prevalence of VI among the staff of the Eye Centre of the KBTH was 9.5 %, all due to refractive errors. Other known causes of avoidable visual impairment and blindness encountered were glaucoma (14.3 %), macular scar (1.2 %) and cataract (1.2 %), all asymptomatic. Routine eye screening should be part of periodic medical examination for employees.

**Funding:**

None declared

## Introduction

Visual impairment (VI) is a global health problem.^1^ VI includes both low vision and blindness. Low vision is defined as visual acuity of less than 6/18 but equal to or better than 3/60, or a corresponding visual field loss to less than 20°, in the better eye with the best possible correction. Blindness is defined as visual acuity of less than 3/60, or a corresponding visual field loss of less than 10°, in the better eye with the best possible correction.^2^

An estimated 314 million people are visually impaired, of which 45 million are blind.^1^ Eighty per cent of the global visual impairment is avoidable, which is either preventable or treatable.^1^ About 90 % of the world's visually impaired and blind live in low-income settings.^1^,^3^

Globally the major causes of blindness are cataract (39 %), uncorrected refractive errors (18 %), glaucoma (10 %), age-related macular degeneration (7 %), corneal opacity (4 %), diabetic retinopathy (4 %), trachoma (3 %), eye conditions in children (3 %), and onchocerciasis (0.7 %).^1^ The major causes of visual impairment, worldwide as reported by WHO, are uncorrected refractive errors (URE) (123.7 million people) and cataract (65.2 million).^4^

Uncorrected refractive errors (URE) are the main causes of moderate and severe visual impairment, and cataracts remain the leading cause of blindness in middle- and low-income countries.^3^ In Sub-Saharan Africa generally and in Ghana specifically, the causes of avoidable blindness are primarily cataract (50 %) and glaucoma (15 %).^5^

Visual impairment has significant socioeconomic implications and decreased quality of life (QoL), especially in those aged over 40 years.^6^ The burden is greater in low and middle-income countries, in older populations, women, and rural populations.^4^ VI has both immediate and long term consequences, including lost educational and employment opportunities, lost economic gain for individuals, families and societies, and impaired quality of life.^6^ Though VI and blindness due to cataract and refractive errors can be treated by surgery and spectacles, respectively, blindness due to glaucoma is irreversible.^1^,^7^ Eyecare workers continue to educate the public but may rarely have their eyes examined. Some members of staff of KBTH have come with severe visual impairment and blindness from glaucoma and proliferative diabetic retinopathy because they were unaware, they needed to have periodic exams or were too busy to do so. These issues necessitated the need to determine avoidable and unavoidable causes of visual impairment among the staff of the Eye Centre at KBTH, a tertiary eye centre, and institute treatment where necessary.

The study hypothesized that at least thirty members of staff of the Eye Centre would be visually impaired, and at least ten will be diagnosed with glaucoma. This hypothesis was based on previous studies in the Ghanaian population where Ansah et al. established the prevalence of VI to be 29.2% in their study population.^8^ Ntim-Amponsah et al. had also established in their populationbased study, the prevalence of glaucoma in patients 30 years and above and in patients 40 years and above to be approximately 8% and 9%.^9^

## Methods

### Study Design

This was a cross-sectional study of all members of staff of the Eye Department KBTH, Accra, Ghana.

### Study Period

From October 2016 to 30th March 2017.

### Study site

The Outpatient clinic, Eye Centre, Korle-Bu Teaching Hospital, Accra, Ghana. The centre has eight full-time ophthalmologists, twenty-seven ophthalmic nurses, eight resident doctors, two optometrists, a research officer and other support staff. The facility is endowed with sub-specialty clinics for Glaucoma, Orbit and Oculoplastics, Neuro-Ophthalmology, Retina, General Clinic, Paediatrics and strabismus, and Low vision. There is a primary screening area made up of 10 consulting rooms run by the ophthalmic nurses.

**Subjects/target population:** All members of staff of the Eye Centre, KBTH.

**Inclusion:** All members of staff of the Eye Centre, KBTH who consented to participate in the study were included.

**Exclusion criteria:** Any member of staff who declined enrolment in the study.

**Sample size:** All 106 members of staff, including investigators.

**Procedure:** Ethical approval was first obtained from the Institutional Review Board (IRB) of the KBTH with reference number IRB/00052/2016. A list of all 106 members was requested from the eye clinic administration and staff were categorized into their various subunits e.g., ophthalmologists, optometrists, administrators, and orderlies. We created groups of not more than 6 members with a mix of various categories of workers. This grouping was done in a convenient manner depending on the schedule of staff members and their availability.

All examinations were planned to ensure that the normal running of the clinic was uninterrupted. Not more than two staff members from any of the units of the Eye Centre were examined together daily. Two ophthalmic nurses in the study team, EB and DF coordinated the flow of members of staff. After counselling and written informed consent, they recorded demographic (age, sex) and clinical (history of symptoms, ophthalmic, and other medicals) data of participants using a predesigned data collection form. Visual acuity in the right eye followed by the left eye was then checked using the Logarithm of Minimum Angle of Resolution (LogMAR) test type. After visual acuity assessment and documentation, examinations were performed and documented in the following order:

1. Ocular alignment was assessed using a pen torch.
2. Eye movements were checked in all directions of gaze.
3. Adnexa and anterior segment of each eye were examined using a slit lamp binocular microscope (Haag-Streit AG, model-BQ 900, SN-05657 Switzerland).
4. Tear Break Up Time (TBUT) was assessed. To perform the TBUT, the participant was positioned behind the slit lamp microscope, a drop of fluorescein 2 % was then instilled into the lower fornix of the right eye in upgaze. The participant was instructed to blink three times to ensure uniform distribution of the fluorescein on the cornea and after that to cease blinking with the eyes closed. The ocular surface was then examined with a broad beam of light with cobalt blue filter in place and a magnification of x10 of the slit lamp. TBUT was recorded using a stopwatch as the time in seconds from the last blink till the appearance of a dark spot on the cornea. This process was then repeated in the left eye. TBUT was recorded for each eye. A TBUT under 10 seconds was considered abnormal.
5. Intraocular pressure was checked for each participant using a Goldman applanation tonometer after instillation of fluorescein 2 % with amethocaine 2 % eyedrops.
6. The fundi of all participants were examined by biomicroscopy using slit lamp binocular microscope (Haag-Streit AG, model-BQ 900, SN-05657 Switzerland), with Volk + 78D or +90D lens and indirect ophthalmoscope (Keeler, UK) with Volk +20D and/or +28D lenses. The fundus examination was done through dilated pupils using a combination of tropicamide 1 %, with phenylephrine 2.5–5 % eye drops (auromide) instilled into each eye and instillation repeated after 15 minutes. The examination was performed after 45 to 60 minutes.
7. The central cornea thickness measurement was after the anterior segment examination with the Pachette 2 ultrasonic pachymeter (model-DGH 550 SN-2151, DGH Technology Incorporated, USA). The pachymeter broke down early in the study period allowing for measurement in only 48 participants.
8. Gonioscopy was performed on all those suspected to have glaucoma using Zeiss goniolens.
9. Visual field test, using a Humphrey visual field analyzer (SITA), was performed for all participants with optic nerve head cup to disc ratio equal to or more than 0.5, and disc asymmetry (defined as the disparity of the optic nerve head cup to disc ratio of 0.2 or more). Confirmation of diagnosis of glaucoma was by two reproducible visual field tests.
10. Refraction was performed for all participants whose uncorrected visual acuities were less than or equal to 6/9 (0.2 Log MAR) unaided and improved by pinhole testing. Refraction was done with autorefractor KW2000 rev auto ref-keratometer (Kowa American Corp., USA), followed by subjective refraction. Spectacles were prescribed for those who needed them.

Visual acuity was classified according to the International Classification of Diseases -10 (Update and Revision 2006).^10^ Visual impairment was classified as; normal vision (LogMAR 0.0–0.4), mild visual impairment (LogMAR 0.5), moderate visual impairment (LogMAR 0.6–1.0), severe visual impairment (LogMAR 1.1–1.4) and blindness (LogMAR 1.5–1.9).^11^

### Data analysis

Data was collected using Microsoft Excel after each questionnaire had been certified as fully completed by the Principal Investigator. Data were analyzed using Statistical Package for Social Scientists (SPSS) Version 20.0. Continuous numerical data were summarized as Mean and Standard deviation (SD) and categorical data as percentages. Data were presented using frequency diagrams and tables, as appropriate. To prove significant outcomes, Chi-square was used to compare proportions at 0.05 significance level.

## Results

Eighty-four (79.3 %) staff members participated in this study. Most of the participants were females (54, 64.3 %). The age range was 23–60 years with a mean of 35.8±9.9 years (mean ± SD) (Table 1).

**Table 1:** Demographic profile and classification of visual impairment among Eye Centre staff

Demographic profile	Classification of VI		P- value	Total N (%)
	NVI N (%)	MVI N (%)		
**Sex** **Female** **Male** **Total**	49(90.7) 27(90.0) 76(90.5)	5(9.3) 3(10.0) 8(9.5)	0.0001	54(100.0) 30(100.0) 84(100.0)
**Age group** **≤ 30** **31–40** **41–50** **≥51** **Total**	28(90.3) 30(96.8) 10(83.3) 8(80.0) 76(90.5)	3(9.7) 1(3.2) 2(16.7) 2(20.0) 8(9.5)	0.0001	31(100.0) 31(100.0) 12(100.0) 10(100.0) 84(100.0)
**Co-morbid conditions** **Hypertension** **Diabetes** **Mellitus** **Asthma**				7(8.3) 1(1.2) 2(2.4)

VI = Visual impairment, NVI = No Visual impairment, MVI = Mild Visual Impairment, P-value for significant difference between males and females = 0.294. Prevalence of mild visual impairment = 9.5%. The mean age (Mean ± SD) was 35.8±9.9.

Prevalence of visual impairment (mild) was 9.5 % (8/84) all due to uncorrected refractive error [hyperopia -6 (7.1 %), and myopia -2 (2.4 %)]. There was a statistically significant difference between participants without visual impairment and those with mild visual impairment (pvalue = 0.0001) (Table 1). Other known causes of VI encountered included glaucoma (primary open angle), found in 12 (14.3 %) participants, all newly diagnosed; unilateral macular scar of unknown cause in 1(1.2 %) participant and 1(1.2 %) with sutural cataract. However, these were not visually significant.

The prevalence of known causes of avoidable visual impairment from the study was 26.2 % (Table 2).

**Table 2:** Ocular morbidities, prevalence, and common causes of avoidable visual impairment among Eye Centre staff

Ocular morbidities	N (%)
**Dry eyes**	22 (26.2)
**Lid abnormalities**	9 (10.7)
**Pinguecula**	3 (3.6)
**Pterygium**	2 (2.4)
**Allergic conjunctivitis**	1 (1.2)
**Keratitis**	1 (1.2)
**Total**	38 (45.2)
**Cause of VI**	N (%)
**Glaucoma**	12(14.3)
**Macular scar**	1(1.2)
**Refractive error**	8(9.5)
**Cataract (sutural)**	1(1.2)
**Total**	22(26.2)

VI= Visual Impairment. Prevalence of glaucoma = 14.3%. Lid abnormalities = [blepharitis- 7 (8.3 %); meibomianitis- 2 (2.4 %)]. Eight (9.5 %) had refractive error [hyperopia - 6 (7.1 %), and myopia – 2 (2.4%)].

The mean intraocular pressure was 15.9±3.6 mmHg and 15.4±3.3 mmHg for right and left eyes, respectively. The mean central corneal thickness recorded was 526.1±32.6 µm and 528.2±32.6 µm for right and left eyes, respectively. The mean cup-to-disc ratio for both right and left eyes was 0.4±0.2 (Table 3).

**Table 3:** Anterior and posterior segments features in study participants

Parameter	Highest	Lowest	Mean
**CCT(N=48)** **Right eye (µm)** **Left eye (µm)**	589 594	447 469	526.1±32.6 528.2±32.7
**IOP(N=84)** **Right eye (mmHg)** **Left eye (mmHg)**	24 23	6 6	15.9 ± 3.6 15.4 ± 3.3
**Vertical cup disc ratio (N=84)** **Right eye** **Left eye**	0.9 0.9	0.1 0.1	0.4 ± 0.2 0.4 ± 0.2
**Cornea TBUT** **Right eye (seconds)** **Left eye (seconds)**			9.0±2.7 8.9±2.6
**Abnormalities** **Lid** **Lens** **Cranial nerve**			9 (10.7) 1 (1.2) 9 (10.7)

Cranial nerve abnormalities (RE II=7, LE II=7, LE VII=1, RE IX=1), IOP = Intraocular pressure, CDR = cup-to-disc ratio, CCT= Central cornea thickness, TBUT = Tear Break-Up Time (in seconds). Lid abnormalities = [blepharitis- 7 (8.3 %); meibomianitis-2 (2.4 %)]. Lens abnormality = [asymptomatic sutural cataract- (1.2 %)]. Cranial nerve abnormalities = [7 (8.3 %) had both RE II and LE II cranial nerve abnormalities all due to glaucoma; 2 (2.4 %) had LE VIII and RE IX cranial nerve abnormalities respectively, with causes not established].

The commonest ocular morbidity was Dry Eye in 22 (26.2 %). The mean tear break-up time for right and left eyes were 9.0±2.7 seconds and 8.9±2.6 seconds, respectively (Table 3). Eye lid abnormalities such as blepharitis and meibomianitis were found in 9 (10.7 %) [blepharitis-7 (8.3 %); meibomianitis-2 (2.4 %)]. Cranial nerve abnormality was found in 9 (10.7 %) of the participants. Out of the 9 participants with cranial nerve abnormalities, 7 (8.3 %) had both RE II and LE II cranial nerve abnormalities all due to glaucoma, and the other 2 (2.4 %) participants had LE VIII and RE IX cranial nerve abnormalities, respectively, with causes not established (Tables 2 and 3).

Out of the 37 participants who had their visual fields plotted, 5 (13.5 %) had a nasal step, 2 (5.4 %) had paracentral scotoma, 4 (10.8 %) had non-specific scotomas, and 20 (23.8 %) had normal visual fields findings (Table 4).

**Table 4:** Visual fields defects encountered in the participants

Defect	Unilateral N (%)	Bilateral N (%)	Total N (%)
**Edge scotoma**	1 (2.7)	-	1 (2.7)
**Inferior scotoma**	1 (2.7)	-	1 (2.7)
**Superior scotoma**	3 (8.1)	-	3 (8.1)
**Nasal step**	2 (5.4)	3 (8.1)	5 (13.5)
**Non-specific scotoma**	4 (10.8)	-	4 (10.8)
**Paracentral scotoma**	2 (5.4)	-	2 (5.4)
**Arcuate scotoma**	1 (2.7)	-	1 (2.7)
**Normal fields**	-	20 (54.1)	20 (54.1)
**Total**	14(37.8)	23(62.2)	37 (100.0)

The commonest co-morbid condition in participants was hypertension, 7 (8.3 %) (Table 1). Family history of glaucoma was recorded among 4 (4.8 %) participants. None of these has had any screening for glaucoma prior to this current study. Six (7.1 %) participants had family history of blindness with the cause of blindness unknown and 2 (2.4 %) had family history of hypertension.

Interventions provided for treatable causes of visual impairment included counselling of affected participants, initiation of drug treatment and referral to the glaucoma clinic for further management and follow up for those diagnosed with glaucoma, refraction and provision of spectacles for 8 (9.5 %) participants with uncorrected refractive errors. Topical antihistamines were provided for those with allergic conjunctivitis, and lid hygiene and warm compression advised for those presenting with blepharitis and meibomianitis. The latter were prescribed with initial doses of ocular lubricants and antibiotic ointments. Participants with pterygia and pinguecula were prescribed with lubricant eye drops.

## Discussion

This study provides baseline data on the prevalence and causes of visual impairment (VI) in staff of an eye centre in a tertiary hospital in Ghana, mostly avoidable causes. To the best of our knowledge, this is the first published data on VI among staff of an eye centre in Ghana or the West African sub-region. This current study's prevalence of avoidable visual impairment was 9.5 % (all mild) and due to uncorrected refractive error. Other known causes of avoidable VI and blindness encountered in this study were glaucoma (Primary Open Angle Glaucoma) (14.3 %) and cataract (1.2 %) but were asymptomatic.

The prevalence of visual impairment found in this current study was lower than that established in other Ghanaian studies by Ansah (28.2 %)^8^ and Budenz *et al.* (17.1 %).^12^ It was, however, higher than the 2 % recorded in Wenchi District.^13^ The lower rate of visual impairment in this study as opposed to the findings by Ansah^8^ in Juaben may be due to different sampling methodology and sample size (84 in this current study versus 1198 in the Juaben study). Our current study was a hospital-based cross-sectional type, whereas the study by Budenz *et al.* in Tema was a population-based study. Other Ghanaian studies have reported visual impairment in the elderly of 22 %^14^ and 17 %^15^, respectively. The prevalence of VI in this current study corroborates findings from a cross-sectional study involving over a thousand patients attending the same eye centre (KBTH eye clinic) by Ntim-Amponsah *et al.*, that demonstrated a rate of 10 % prevalence of VI.^16^

All the visually impaired in the current study had a refractive error as the underlying cause. Other hospitalbased studies conducted by Maake in South Africa and Ntim-Amponsah *et al.* in Ghana have found refractive error as the commonest cause of VI, with prevalence of 38% and 44%, respectively.^6^,^16^ In the Nigerian national visual impairment study, refractive error accounted for 57% of visual impairment.^17^

Other causes of VI encountered in other studies include cataract and glaucoma.^6^ Previous studies in Ghana have found uncorrected refractive error and glaucoma as major causes of visual impairment and blindness, respectively.^5^,^13^ Although this current study found a prevalence of glaucoma to be 14.3%, none of the affected participants had VI or blindness. Previous studies had demonstrated a higher prevalence of glaucoma (18.3 %) and refractive error (21.6 %)^17^ compared with results from this current study. Other studies from Ghana, all population-based types, reported varying results compared with the current study: The Akwapim South survey found a lower prevalence of 8.5 % of people ≥40 years and 7.7 % aged ≥30 years had glaucoma^9^, however, a recent population-based survey found a higher prevalence of glaucoma which accounted for 19.4 % of blindness in Ghana.^5^ These disparities may be explained in part by the different study types. As previously indicated, though glaucoma was the commonest pathology picked up in this study (14.3 %), fortunately none of the participants was blind or visually impaired from the disease and timely interventions were instituted by way of topical glaucoma drugs and close monitoring. The challenges with glaucoma, unlike refractive error and cataract is the fact that visual impairment or blindness from glaucoma is irreversible.^18^,^19^ People with glaucoma are asymptomatic in the early stages and thus early diagnosis to avoid visual impairment should be through routine eye screening.^20^,^21^ Finding the patient while there is still some useful vision to save is one of the major challenges in the management of glaucoma in Africa.^22^

The prevalence of cataract in this current study conducted in a working population (retirement age of 60 years) was very low (1.2 %). The low prevalence of cataract and specifically the absence of senile cataract may be due in part to the relatively younger age of the staff that were examined, mean age of 35.8±9.9 years. A Ghanaian study in Wenchi however found 39.0 % of their study participants with cataract.^13^ This disparity is not surprising since the etiology of cataracts, though multifactorial, are mostly senile occurring usually in patients aged over 50 years.^23^,^14^,^15^ The only participant with a cataract had a sutural cataract. Participant was unaware of this which suggests that she had never had her eyes checked even though she worked in an eye clinic.

There was no significant difference between visual impairment in males and females (p-value = 0.294) in this present study, a finding that supports findings from other studies that the disparity between male**s** and females is slightly lower in sub-Saharan Africa than in the high income regions.^24^ This observation is however not supported by other studies which demonstrated that both age and gender affect VI and that, prevalence increases with age^5^,^8^,^14^,^15^ with women having a significantly higher risk of developing VI than men in most regions of the world.^5^,^8^,^15^,^24^,^25^

The other significant ocular morbidity found in this current study was dry eye (22.6 %). This is similar to findings from a Ghanaian study which reported up to 20 % of cases seen at their out-patient clinic daily presented with dry eye,^26^ as well as other studies. 27 Dry eye syndrome (DES) is recognized as a growing public health disease and is one of the most common causes of ophthalmological consultation.

Surgical residents as well as other staff of the eye clinic may have a higher risk of developing DES because of frequent use of operating microscopes, slit lamps and other high-tech equipment in an eye clinic. There may be the need for further studies on dry eyes in this population. The study hypothesized that at least 30 members of staff of the Eye Centre will be visually impaired. Findings from the study however, showed that 8 (9.5 %) participants had mild visual impairment. Another hypothesis was that at least 10 of the members of staff of the Eye Department will be diagnosed as having glaucoma, with findings from the study showing that 12 (14.3 %) had glaucoma (all newly diagnosed).

Eye health workers in a tertiary eye care facility do not face any barriers to receiving eye care since these services are readily accessible to them. There is the need to promote eye health education among these health workers.

The findings from this study strengthen the need for a hospital policy for routine medical screening of staff to diagnose early, not only avoidable causes of visual impairment and blindness, but also other systemic medical conditions that can cause morbidity and mortality if not detected early. This can also be done just to satisfy management as part of conditions of employment, a reason that compels some staff to undertake periodic health examination in some set-ups as evidenced by a study in Illorin, Nigeria.^28^

## Conclusion

The prevalence of visual impairment among staff of the Eye Centre of the KBTH was 9.5 % all due to refractive errors. Other known causes of avoidable visual impairment and blindness encountered were glaucoma (14.3 %), macular scar of unknown aetiology (1.2 %) and cataract (1.2 %).
